# Efficient inhibition of cervical cancer by dual drugs loaded in biodegradable thermosensitive hydrogel composites

**DOI:** 10.18632/oncotarget.22965

**Published:** 2017-12-06

**Authors:** Shan Xu, Xiaobo Du, Gang Feng, Yu Zhang, Jie Li, Binwei Lin, Linglin Yang, Shaozhi Fu, Jingbo Wu

**Affiliations:** ^1^ Department of Oncology, Mianyang Central Hospital, Mianyang 621000, Sichuan Province, China; ^2^ Department of Oncology, The Affiliated Hospital of Southwest Medical University, Luzhou 646000, Sichuan Province, China

**Keywords:** cervical cancer, cisplatin, paclitaxel, thermosensitive hydrogel, micelles

## Abstract

**Background and Purpose:**

We aimed to explore the anti-tumor effect and mechanism of the combination of cisplatin (DDP)-containing thermosensitive hydrogel (PEG-PCL-PEG, or PECE) and paclitaxel (PTX)-loaded MPEG-PCL polymeric micelles called PDMP on human cervical carcinoma (HeLa) cell. In our previous studies, we found that PDMP *in situ* treatment of lung cancer will be liable to have potential in Lung cancer patients.

**Results:**

Compared with other treatments, PDMP was most effective in prolonging survival time (*P* < 0.05), inhibiting tumor growth (*P* < 0.05), decreasing expression of CD133 (*P* < 0.05), CD31 (*P* < 0.05), and aldehyde dehydrogenase 1 (ALDH1) (*P* > 0.05), inducing G1 phase arrest (*P* < 0.05), increasing the apoptosis rate (*P* < 0.05), and in expressing ATM and γ-H2AX (*P* < 0.05).

**Conclusions:**

PDMP is regarded as a promising anti-tumor reactant, when it comes to the treatment of cervical carcinoma.

**Methods:**

we used a xenograft cervical cancer model to verify the anti-tumor activity of PDMP and to explore its mechanism of action. Mice were intratumorally administered with NS, PECE, PTX+DDP or PDMP. After two days of treatment, three mice per group were sacrificed and tumor tissue was harvested. Levels of histone H2AX phosphorylation (γ-H2AX) were determined by immunohistochemistry and ataxia telangiectasia mutated(ATM) protein levels were measured by western blot analysis. In addition, it would sacrifice each of group of three mice through 10 days’ treatment, what’s more, it would harvest tumor by virtue of flow cytometry and immunohistochemical analysis. It would like to use there maining mice to analyze tumor growth and survival. The remaining mice were analyzed for tumor growth and survival.

## INTRODUCTION

It is said that cervical cancer is the most generaltype of cancer in females worldwide and in developing countries [1]. In China, approximately 133,000 new cervical carcinoma patietns are reported annually [2]. Due to its biological characteristics, cervical cancer cells easily contribute to local invasion and recurrence, therefore local treatment is the preferred treatment approach [3]. The standard treatment for locally advanced cervical cancer is radiotherapy or surgery, however treatment results are often disappointing. As a neoadjuvant approach, chemotherapy has had a beneficial impact on the survival of cervical cancer patients, has increased tumor-free survival rates, and may allow surgical therapy in patients who initially would not be considered for tumor resection [4]. Recent studies have demonstrated that combination neoadjuvant chemotherapy with paclitaxel (PTX) and cisplatin (DDP) can reduce tumor volume and improve the operative rate with low toxicity, meaning that this therapeutic schedule can improve the long-term survival of patients with cervical cancer [5, 6]. Even though, it has made essential progress in treatingsome cancers, the vital hypersensitivity reactions associate with chemotherapy with PTX and DDP, the therapeutic benefits of their using are liable to be offset by hematologic toxicity and neurotoxicity [7]. Thus, innovative approaches for treatments with limited side effectsare of the utmost importance.

Over the past few decades, injectable *in situ* hydrogels haveattracted considerable interest as vehicles for drug delivery, especially in anticancer chemotherapy. It is not difficult to manage the Injectable hydrogels, biocompatible, improve patient compliance, lead to a high regional drug focus, and have low systemic toxicity [8, 9]. Prior to administration, these hydrogels are aqueous solutions, however once injected, they rapidly turn into gel owing to one orstimuli’s combination, such as pH change, temperature modulation, or solvent exchange [10]. Polymeric nanoparticles (NPs) have also attracted a lot of attention which is regarded as potential drug delivery systems, because they are able to effectively deliver a drug to a target site, escaping from the vasculature by virtue of leaky endothelium, improving therapeutic effects, and reducing side effects [11].

In our former research [12], there was a novel biodegradable and injectable gel-forming hydrogel composite that was prepared by us called PDMP, which is composed of MPEG-PCL/PTX NPs and a PECE/DDP hydrogel. PDMP turns out that it has great potential in curing lung cancer, which refers to a novel, biodegradable, temperature-sensitive and injectable *in situ* drug delivery system. Considering the biological characteristics of cervical cancer, and the position and anatomical structure of the cervix, we believe the PDMP hydrogel composite may be ideal *in situ* treatment for cervical cancer. Here, we used the PDMP hydrogel composite in a cervical cancer model to verify the anti-tumor activity of PDMP, and to comprehensively investigate its antineoplastic mechanism.

## RESULTS

**Figure 1:**
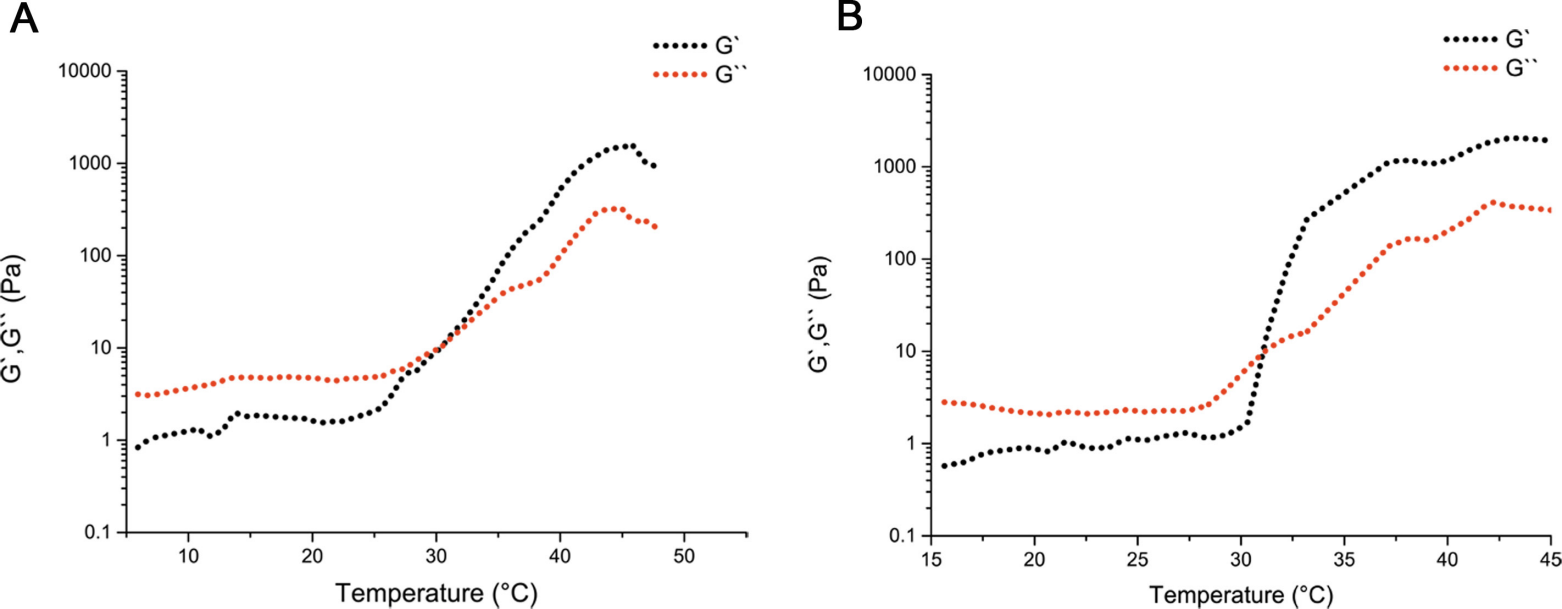
Thermal sensitivity of the injectable PDMP hydrogel composite Thermal sensitivity of the PECE hydrogel composite (**A**) and the PDMP hydrogel composite (**B**) was examined by oscillatory rheological tests.

MPEG-PCL and MPEG-PCL/PTX micelles were successfully synthesized according to previously reported studies [13, 14]. According to the HPLC measurements, the DL and EE of the MPEG-PCL/PTX micelles were 3.97 ± 0.05% and 99.05 ± 0.78%. PECE was synthesized by ring-opening copolymerization of ɛ-CL initiated by PEG using Sn(Oct)_2_ as catalyst, which was reported previously [15]. Next, the mixing MPEG-PCL/PTX micelles and the DDP solution into the PECE solution could synthesize the PDMP hydrogel. Those oscillatory rheological tests could evaluate the PECE copolymer’s mechanical properties and thermo-sensibility. The evaluation ofthe kinetics of hydrogel formation measured to the storage modulus (G’) and loss modulus (G’’).Figure 1A and 1B show the differences between G’ and G’’ in the blank PECE hydrogel and the PDMP hydrogel composite independently. The composite with a low G’ and G’’ and the temperature was less than 30°C, which indicated that the composite means the flowing fluid which has low viscosity. If the temperature was beyond 30°C, the samples would turn a liquid into an elastic gel-like substance at the crossover point of G’ and G’’. Moreover, thermo-sensitivity was displayed in the PDMP hydrogel composite, confirming the blank PECE hydrogel’s consequences.

**Figure 2:**
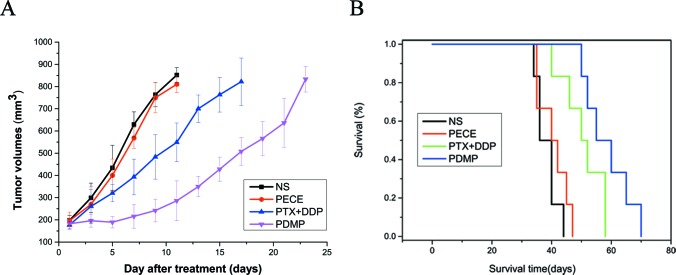
PDMP inhibited tumor growth in a subcutaneous HeLa model (**A**) Suppression of subcutaneous tumor growth by PDMP in mice; (**B**) Mouse survival curve for each group; PTX, paclitaxel; DDP, cisplatin; PDMP, mixed MPEG-PCL/PTX micelles with DDP-loaded PECE hydrogel.

To evaluate the efficacy of PDMP treatment on HeLa xenografts in mice, tumor volume and median survival time were measured and plotted follow in treatments. The tumor size and shape of the tumor in various groups are shown in Supplementary Figure 1. Tumor growth curves are shown in Figure 2A, and reveal that the PDMP treatment was rather too effective at controlling tumor development when it was compared with the other teams (*P <* 0.05 in all cases). In the PECE group, an anti-tumor effect was not observed. The median survival time ofthe PDMP group (55 days)was significantly longer than the PTX+DDP group(50 days, *P <* 0.05), PECE group (40 days, *P <* 0.05), and NS group (36 days, *P <* 0.05) (Figure 2B). In terms of the tumor growth and survival time between the NS and PECE groups, it didn’t observe the dramatically differences. (*P >* 0.05, in all cases). In addition, as is shown in Table 1, PDMP treatment alone resulted ina TGD of 8 days, which was significantly different to the PTX+DDP group (4.7 days, *P <* 0.05), PECE group(0 days, *P <* 0.05), and NS group (0 days, *P <* 0.05). Those consequencessuggested that PDMP could not only effectively suppressed tumor development, but also the tumor-bearing mice’s survival could be prolonged.

**Table 1:** Tumor growth delay in cervical cancer after different treatments

Groups	T4v0 (days)	TGD (days)
NS	11.3	0
PECE	11.3	0
PTX+DDP	16	4.7
PDMP	19.3	8

PTX, paclitaxel; DDP, cisplatin; PDMP, mixing MPEG-PCL/PTX micelles with DDP-loaded PECE hydrogel.

**Figure 3:**
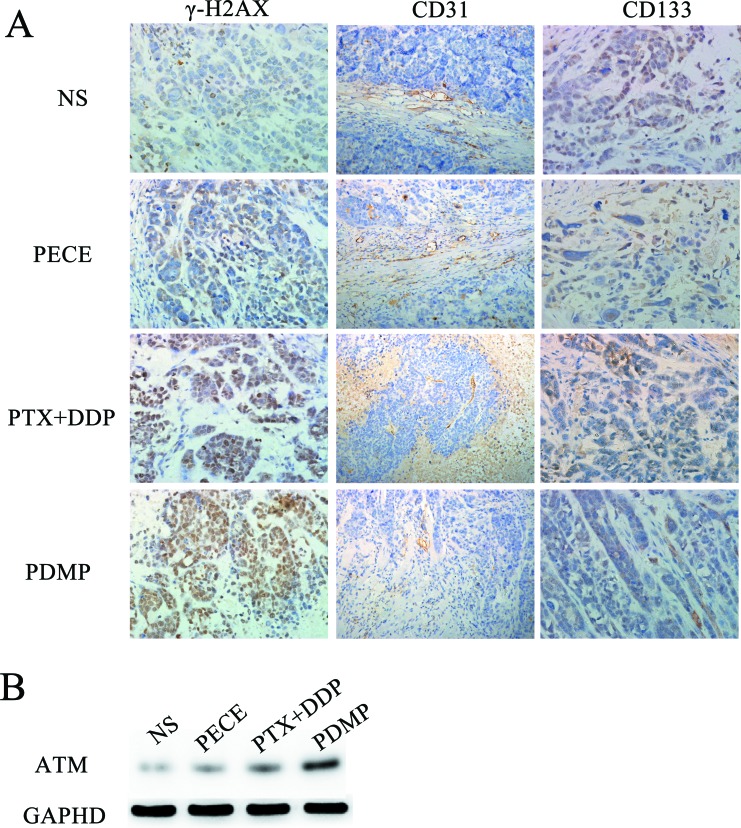
Immunohistochemical analysis of γ-H2AX, CD133 and CD31 and Western blot of ATM in xenografts from mice in various groups (**A**) Immunohistochemical analysis of γ-H2AX, CD133 and CD31 in xenografts from mice in various groups; (**B**) Western blot of ATM in xenografts from mice in various groups; PTX, paclitaxel; DDP, cisplatin; PDMP, mixing MPEG-PCL/PTX micelles with DDP-loaded PECE hydrogel.

**Figure 4:**
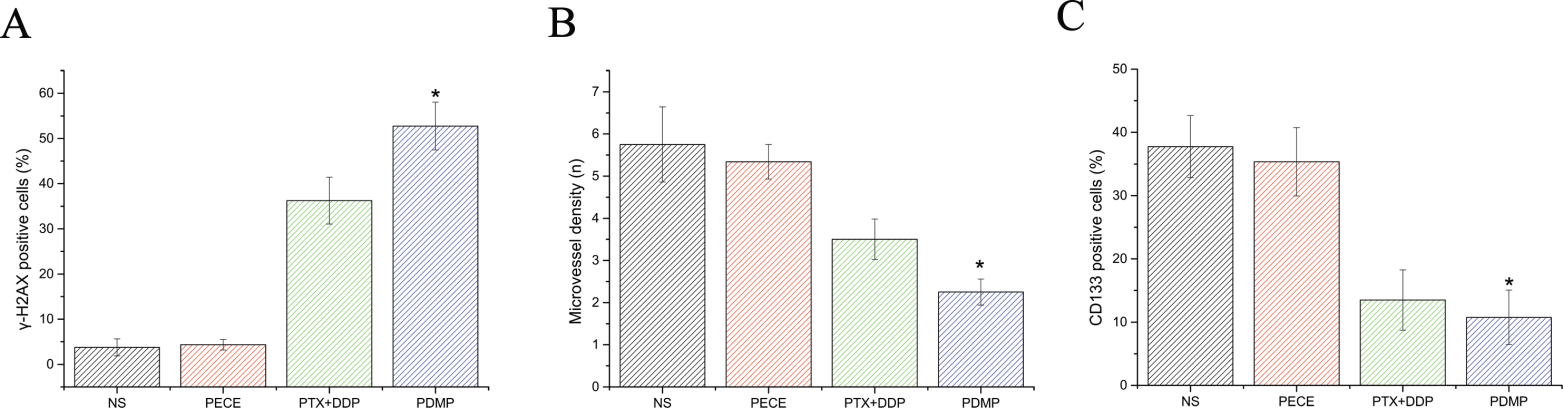
Quantitative analysis of γ-H2AX, CD133 and CD31 in xenografts from mice in various groups (**A**) Quantitative analysis of γ-H2AX in xenografts from mice in various groups; (**B**) Quantitative analysis of CD31 in xenografts from mice in various groups; (**C**) Quantitative analysis of CD133 in xenografts from mice in various groups; ^*^p < 0.05; PTX, paclitaxel; DDP, cisplatin; PDMP, mixed MPEG-PCL/PTX micelles with DDP-loaded PECE hydrogel.

The anti-tumor effect of PDMP in mouse xenografts can be determined by assessing the expression of tumor-associated γ-H2AX, which represents the level of DNA damage [16] (Figure 3A). As is shown in Figure 4A and Table 2, observing tumor- associated tissue in the PDMP group, it could find that there is an increased number of γ -H2AX positive cells (52.75 ± 5.31%) compared to the PTX+DDP-treated group (36.25 ± 5.18%, *P <* 0.01), PECE group (4.34±1.15%, *P <* 0.01), and the NS group (3.75 ± 1.89%, *P <* 0.01). It didn’t find the important differences between the NS and blank PECE groups (*P >* 0.05). The expression of CD31, an endothelial cell surface molecule that can be used to visualize MVD, was evaluated by immunohistochemistry [17] (Figure 3). As seen in Figure 4B and Table 2, fewer immunoreactive microvessels were detected in tumor sections derived from the PDMP-treated mice (2.25 ± 0.31), when compared to PTX+DDP-treated (3.5 ± 0.48, *P <* 0,05), PECE-treated (5.34 ± 0.41, *P <* 0.01), or NS groups (5.75 ± 0.89, *P <* 0.01). The effect on tumor proliferation was assessed by immunohistochemical analysis of CD133 (Figure 3), which is a molecular marker for cancer stem cells (CSC) [18, 19]. As shown in Figure 4C and Table 2, a decrease in CD133-positive cells was observed in tumors derived from the PDMP-treated mice (10.75 ± 4.31%), when compared to PTX+DDP-treated (13.5 ± 4.78%, *P <* 0.05), PECE-treated (35.34 ± 5.41%, *P <* 0.01), and NS mice (37.75 ± 4.89%, *P <* 0.01). Overall, these results suggest that PDMP treatment significantly increased the number of DNA breaks, decreased MVD, and inhibited CSC, indicating PDMP effectively inhibited tumor growth.

**Table 2:** The immunohistochemical analysis ofγ-H2AX, CD31, CD133 expression in xenografts from mice in various groups

Group	γ-H2AX (%)	CD31 (*N*)	CD133 (%)
PDMP	52.75 ± 5.31	2.25 ± 0.31	10.75 ± 4.31
PTX+DDP	36.25 ± 5.18	3.5 ± 0.48	13.5 ± 4.78
PECE	4.34 ± 1.15	5.34 ± 0.41	35.34 ± 5.41
NS	3.75 ± 1.89	5.75 ± 0.89	37.75 ± 4.89

PTX, paclitaxel; DDP, cisplatin; PDMP, mixing MPEG-PCL/PTX micelles with DDP-loaded PECE hydrogel.

ATM is one of the earliest and most sensitive responses to DNA damage [20]. To further investigate the DNA damage in our treatment groups, ATM protein expression was evaluated by western blot analysis (Figure 3B). We found that higher levels of ATM were detected in the PDMP-treated group compared with other groups.

**Figure 5:**
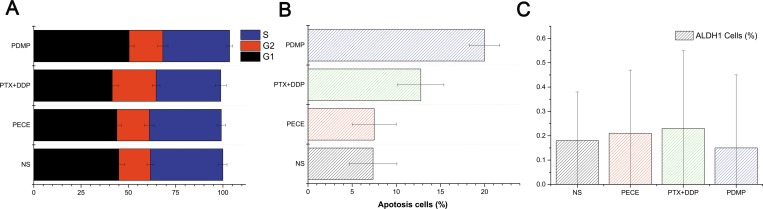
Flow cytometric analysis of tumor tissue from mice that received different treatments (**A**) Quantitative analysis of the percentage of cells in G1, S, G2/M phase in xenografts from mice in various groups; (**B**) Quantitative analysis of the percentage of apoptotic cells in xenografts from mice in various groups; (**C**) Quantitative analysis of the percentage of ALPH1 in xenografts from mice in various groups; PTX, paclitaxel; DDP, cisplatin; PDMP, mixed MPEG-PCL/PTX micelles with DDP-loaded PECE hydrogel.

To identify the mechanism associated with PDMP-related anti-tumor effects in mice, cell apoptosis, cell cycle redistribution, and CSC were evaluated by flow cytometry. As is shown in Figure 5A, Supplementary Figure 2 and Table 3, PDMP treatment caused an accumulation of cells in the G1 phase (50.4 ± 2.84%), compared with PTX+DDP treatment (41.48 ± 3.23%, *P <* 0.05), PECE treatment (43.94 ± 2.48%, *P <* 0.05), and the NS group (44.94 ± 2.91%, *P <* 0.05). However, no significant differences were observed in the percentage of cells in S or G2 phases among these groups (*P >* 0.05, in all cases). In addition, flow cytometric analysis was performed on murine tumor tissues in order to determine whether PDMP could promote cell apoptosis. As seen in Figure 5B, Supplementary Figure 2 and Table 3, the PDMP group (20 ± 1.73%) showed a significant increase in the percentage of cell death compared to the PTX+DDP group (12.78 ± 2.62, *P <* 0.05), PECE group (7.5 ± 2.5, *P <* 0.05) and NS group (7.37 ± 2.7, *P <* 0.05). ALPH1 was used as a CSC marker in HeLa cells, as it represents cell proliferation [21]. As shown in Figure 5C, Supplementary Figure 2 and Table 3, PDMP-treated mice had a reduced percentage of ALPH1 (0.15 ± 0.3%) compared to the PTX+DDP-treated group (0.16 ± 0.45%), PECE-treated group (0.21 ± 0.26%), and the NS group (0.18 ± 0.2%). However, no significant differences were observed between the treatment groups (*P >* 0.05, in all cases).

**Table 3:** The flow cytometry analysis of cell cycle redistribution, apoptosis and Aldehyde dehydrogenase 1 (ALDH1) in xenografts from mice in various groups

Group	G1 (%)	G2 (%)	S (%)	Apoptosis (%)	ALDH1 (%)
PDMP	50.4 ± 2.84	17.84 ± 2.7	35.3 ± 1.62	20 ± 1.73	0.15 ± 0.3
PTX+DDP	41.48 ± 3.23	23.19 ± 2.03	34.2 ± 2.98	12.78 ± 2.62	0.16 ± 0.45
PECE	43.94 ± 2.48	17.12 ± 2.5	38.08 ± 2.3	7.5 ± 2.5	0.21 ± 0.26
NS	44.94 ± 2.90	16.66 ± 1.76	38.37 ± 2.17	7.37 ± 2.7	0.18 ± 0.2

PTX, paclitaxel; DDP, cisplatin; PDMP, mixing MPEG-PCL/PTX micelles with DDP-loaded PECE hydrogel.

Among the treatment groups, no adverse effects, such as changes in appetite, feeding, and behavioral change, were observed. To further determine whether PDMP has cytotoxic effects *in vivo*, H&E staining was performed on sections of mouse organs (kidneys, lung liver, spleen, and heart) (Supplementary Figure 3). Sections were observed by two pathologists, who were blinded to the treatment groups. Our results demonstrated that mice treated with PECE and PDMP showed mild symptoms of liver toxicity (ballooning degeneration). We speculate that this may be related to degradation of the polymer. However, other organs did not show any signs of toxicity.

## DISCUSSION

Worldwidely speaking, cervical cancer is the leading female malignancy worldwide and is also a common reason that results inthe death among middle-aged females [1]. Hough it is readily effective to screen for cervical cancer, most women don’t screen regularly to diagnose with cervical cancer [22]. As a consequence, there are about 25 percent of patients who have cervical cancer in the United States displayed with locally advanced disease (stage IIB through IVA in terms of the staging system of the International Federation of Gynecology and Obstetrics) [23]. Approximately 50% of patients presented with locally advanced cancer of the cervix, and the standard treatment was concurrent chemoradiotherapy (CCRT) [24]. However, during this treatment, toxicity occurred, as indicated by myelosuppression, alarge bowel, and rectal side effects. Therefore, due to these side effects, over 25% of patients were forced to terminate treatment [7]. Recent research has shown that the benefits of neoadjuvant chemotherapy for patients with locally advanced cervical cancer superior to CCRT, nevertheless, it with fewer negative impacts [24, 25]. As for locally advanced cervical cancer, Platinum-based neoadjuvant chemotherapy is regarded as a promising treatment choice. Combination therapy using PTX and DDP has been effective in advanced and recurrent cervical cancers with response rates of 40–50%. However, conventional approaches are used to deliver chemotherapeutic agents who fail to complete the desired therapeutic drug concentrations, and systemic chemotherapy is associated with extensive toxicity, including hematological and gastrointestinal side effects . Thus, novel drug delivery systems are necessary to improve the delivery of drugs and to decrease the toxicity of drug during cancer treatment.

As novel biomaterials, thermal-sensitive hydrogels are being designed for *in situ* gel-forming drug delivery and have been successfully used to carry different drugs during disease treatments [26–28]. Considering their biological characteristics, cervical cancer cells easily contribute to local invasion and local recurrence and there is a connection between locoregional recurrence and distant metastasis in cervical cancer. We hypothesized that injectable *in situ* hydrogels are the most suitable drug delivery system for the treatment of cervical cancer, due to the position and anatomical structure of the cervix. In ours former study [12], there is an *in situ* gel-based dual drug delivery system which was prepared by us, PDMP hydrogel, by virtue of the combination of a DDP -containing thermosensitive hydrogel (PECE /DDP) and PTX -loaded MPEG-PCL polymeric micelles (MPEG-PCL/PTX). We investigated PDMP hydrogels in *in vitro* drug release studies using dialysis, *in vitro* cytotoxicity using the MTT assay, and *in vivo* anti-tumor efficacy in a xenograft lung cancer model. Our results demonstrated that PDMP treatment resulted in slower drug release (over 14 days), minor toxicity, effective inhibition of tumor growth, and prolonged survival in a xenograft lung cancer model.

In this study, the PDMP hydrogel’s anti-tumor efficacy was comprehensively investigated in an *in vivo* cervical cancer model. The rheological results indicated that the PDMP hydrogel has similar thermo sensitive properties to PECE. For the *in vivo* tests, when compared to the other treatment groups, the PDMP-treated group showed the greatest efficacy in TGD and had the highest survival rate. To investigate the mechanism of the enhanced anti-tumor effect of PDMP treatment, animals were sacrificed after 2 or10 days of treatment. PECE hydrogels disappeared *in vivo* at day 14 [29], and tumor tissue was harvested for different analysis by different methods, such as immunohistochemistry and flow cytometry.

Treatment with PTX and DDP induced programmed cell death and cell cycle arrest; therefore flow cytometry analysis was performed using mouse tumor tissues to determine whether PDMP could also promote apoptosis and cell cycle redistribution. The results showed that PDMP treatment resulted in more cells in the G1 phase compared with other treatment groups. This cell cycle change may be due to the fact that PDMP can sustainably release low concentrations of PTX, which completely inhibits cell proliferation without arresting cells at mitosis [30]. In addition, a significant increase in the induction of apoptosis was observed in the PDMP-treated group compared to the other treatment groups. These results suggested that, due to the induction of G1 cell phase arrest and enhancement of apoptosis, PDMP contributed to inhibition of tumor growth.

DNA means a significant target of chemotherapeutics and causes damage to DNA, including base damage, sugar damage, SSBs (single-strand breaks)and DSBs (double-strand breaks), what’s more, DSBs contain histone H2AX phosphorylation and lead to cell death [31]. In our study, we evaluated the expression of histone H2AX phosphorylation by immunohistochemistry in mouse tumor tissue following different methods of treatment. Our results showed that PDMP-treated mice showed the highest expression of γ-H2AX compared to the other treatment groups. To determine the extent of DSB damage, we evaluated the expression of ATM by western blot analysis. ATM has a significant influence on observing DNA DSBs and in line with DNA repair, cell cycle arrest, and in the induction of apoptosis [32]. Our results correlated well with histone H2AX phosphorylation, as PDMP-treated mice showed the highest levels of ATM compared to the other treatment groups. Therefore, the anti-tumor effect of PDMP may be due to enhanced DSBs.

It is well known that PTX and DDP show anti-angiogenic activity, therefore we determined the anti-angiogenic activity of PDMP treatment by immunohistochemical analysis of CD31, which is an endothelial cell surface marker that can be used to visualize MVD. We found that the amount of MVD in the PDMP-treated group was significantly lower than the other groups. This indicated that the anti-tumor effect of PDMP may be caused by its anti-angiogenic activity.

Traditionally, anti-tumor treatments are based on the capacity to eliminate the bulk of the tumor cell population and not a small population of CSCs, which are the driving force for tumor growth and metastasis. In this study, we evaluated the expression of the stem cell-related markers CD133 and ALDH1 by immunohistochemistry and flow cytometry. Our results demonstrated that, compared to other groups, PDMP treatment decreased the number of CD133 and ALDH1 positive cells, suggesting the anti-tumor effect of PDMP may be due to the inhibition of CSCs.

To investigate the *in vivo* cytotoxicity of PDMP, H&E staining was performed on liver, lung, kidney, spleen, and heart of treated mice. As demonstrated in our previous study, PDMP treatment was well tolerated [12].

According to our study, PDMP is a promising anti-tumor agent for the treatment of cervical carcinoma. This is based on the fact that PDMP treatment increased PTX and DDP exposure time in tumors (over 14 days), which is similar to a drug delivery pump. Free PTX and DDP, however, were quickly removed from the body, maintaining only a low concentration in the tumor for a relatively short period of time. Therefore, we believe that our thermosensitive PDMP hydrogel forms a stationary gel in the cervix at body temperature, which serves as a drugdepot and a suitable drug delivery system. Thus, PDMP hydrogels may be a more effective treatment method for locally advanced cervical cancer.

Although the results of this study are promising, there are some limitations. Firstly, our study lacks a deeper investigation of a signaling pathway involved in the activity of PDMP. Secondly, we still do not know the degradation pathway of PDMP in the body, and whether the degradation products may cause harm to humans as organic solvents and cross linking agents are used in the hydrogel synthesis. In addition, the drug dose of paclitaxel and cisplatin set in the study were based on the study of free drug. It may not the optimal dose for hydrogel complex. In our further studies, we will use different doses of drugs to obtain the optimal dose for hydrogel complex. Therefore, future studies will be necessary to confirm our results.

## CONCLUSIONS

In this study, a gel-based dual drug delivery system (PEG-PCL-PEG/Cisplatin+MPEG-PCL/Paclitaxel, or PDMP) was prepared and its *In vivo* anti-tumor activity and *In vivo* toxicity were investigated in a mouse model for cervical cancer. The *in vivo* assay showed that compared with other treatments, PDMP was most effective in inhibiting tumor growth, prolonging survival time, decreasing expression of CD31, CD133, and ALDH1, inducing G1 phase arrest, increasing the apoptosis rate, and in expressing ATM and γ-H2AX. In addition, PDMP are well tolerated by the mice. Thus, the results indicated that PDMP is a promising anti-tumor for local treatment of cervical carcinoma.

## MATERIALS AND METHODS

### Materials, cell lines, and animals

Chemicals used in this study included: poly(ethylene glycol) methyl ether (MPEG, Mn = 550 and 2000, Sigma Aldrich, USA), poly(ethylene glycol) (PEG, Mn = 1000, Fluka, USA), ɛ-caprolactone (ɛ-CL, Alfa Aesar, USA), hexamethylene diisocyanate (HMDI, Sigma Aldrich, USA), stannous octoate (Sn(Oct)_2_, Sigma Aldrich, USA), Dulbecco’s modified Eagle’s medium (DMEM, Sigma Aldrich, USA). Methanol and acetonitrile (HPLC grade) were purchased from Kelong Chemicals (Chengdu, China). PTX and DDP were purchased from Chengdu Man Site Biotechnology Co. Ltd. (Chengdu, China). CD31and CD133 polyclonal antibodies were purchased from Bioworld Technology Co. Ltd. (Nanjing, China).

Human cervical carcinoma cells (HeLa)were supplied by the Experimental Medicine Center at the Affiliated Hospital of Southwest Medical University (Luzhou, China). HeLa cells were grown in DMEM medium and supplemented with 10% fetal bovine serum (FBS), penicillin (100 U/mL) and streptomycin (100 μg/mL) at 37°C in 5% CO_2_.

Three-week old female BALB/c nude mice were used for evaluating the effects of *in vivo* anti-tumor tests. All animals were purchased from Chongqing TengXin Biological Technology Co., Ltd. (Chongqing, China)and maintained in a standard specific pathogen-free (SPF) environment. All animals had access to eatingfood and drinking tap water ad libitum. In accordance with Institutional Animal Care and Use guidelines, it can conduct Animal care and experimental procedures.

### Preparation of MPEG-PCL/PTX micelles

Ring-opening polymerization of ε -CL can make a good preparation of MPEG -PCL/ PTX micelles MPEG-PCL copolymer, initiated by MPEG applying Sn(Oct)2 as catalyst, which was presented in former researches [33, 34]. Obviously, the calculation of MPEG and ε-CL was put in a dry glass ampoule under nitrogen and Sn(Oct)2 was added into the reaction vessel under mild agitation. The reaction system was kept at 130°C for 6 hours. The purified MPEG-PCL copolymer was kept in a desiccator before further use.

PTX micelles were prepared by a one-step solid dispersion method [35]. Briefly, one hundred milligrams of PTX and MPEG-PCL copolymer at different ratios were co-dissolved in 5mLof dehydrated alcohol with mild stirring. Then, the solution was evaporated in a rotary evaporator at 60°C. During this process, homogenous coevaporation was obtained, and PTX was distributed in the MPEG-PCL copolymer as an amorphous substance. Subsequently, there maining material was dissolved in normal saline (NS) at 60°C to self-assemble into micelles containing PTX. The PTX micelles were filtered through a 0.22 μm syringe filter(Millex-LG, Millipore Co., USA), and were lyophilized and stored at 4°C before use.

### The MPEG-PCL/PTX micelles’drug-loading and encapsulation efficiency

The DL and encapsulation efficiency (EE) was decided in triplicate through reverse-phase High Performance Liquid Chromatography (RP-HPLC) using a C18 column (4. 6 mm × 250 mm -5 μm, Grace Analysis column).First, the deionized water at 65° could dissolve the PTX micelles for the sake of breaking their packaging, filtered through a 220-nm filter to get anobvious and brief solution, and then measured at 227 nm. Acetonitrile/water (50/50, v/v) was applied as a mobile stagewitha flowing rate of 1. 0 mL /min. DL and EE tocalculatedusethe following equations: (1) DL% = drug / (polymer + drug) ×100%; (2) EE % = actual drug loading / theoretical drug loading×100%

### Preparation of PECE and PDMP hydrogel composite

**Figure 6:**
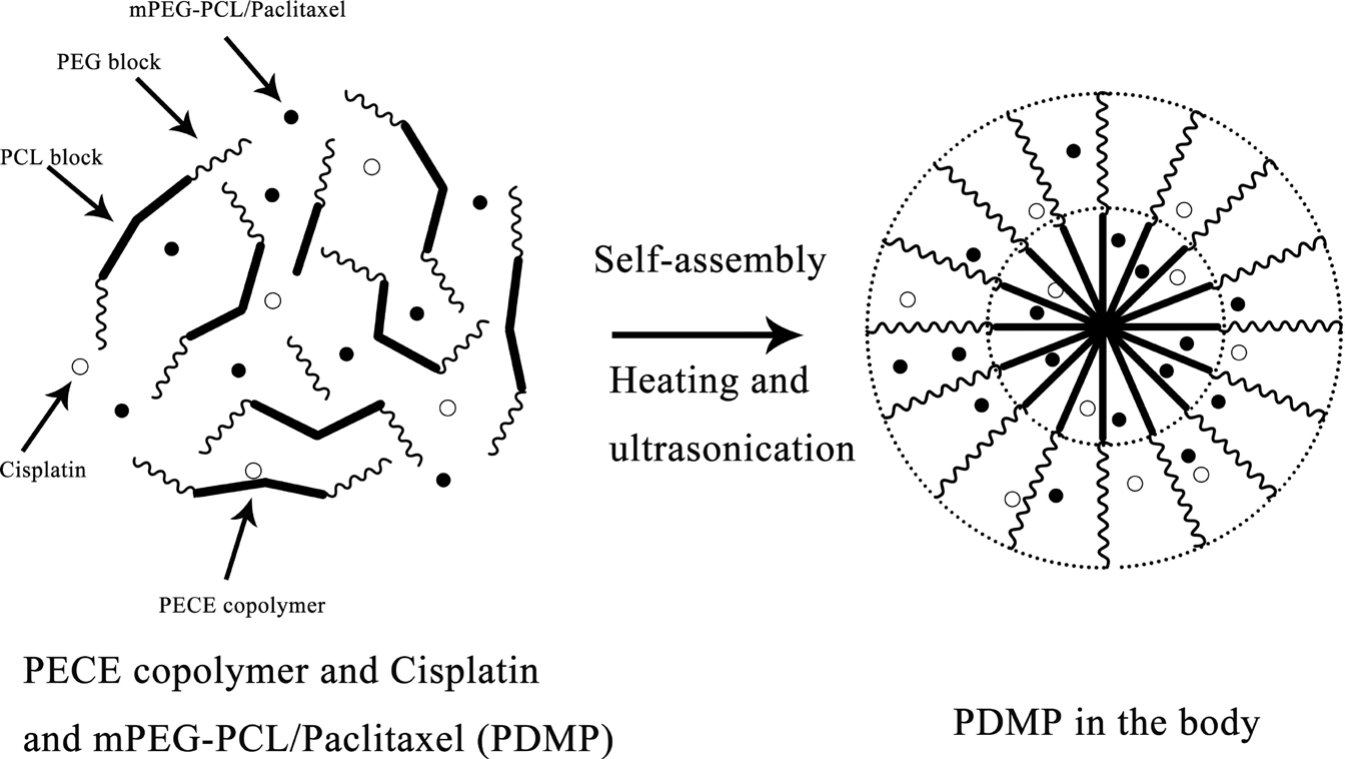
Schematic diagram of the PDMP hydrogel complex

The synthesis and characteristics of the PECE and PDMP hydrogels have been described in previous studies [13, 35]. A schematic diagram of the hydrogel complex is shown in Figure 6. Briefly, PEG-PCL diblock copolymers were synthesized by ring-opening polymerization of ε-CL, initiated by MPEG. Then, by coupling PEG-PCL diblock copolymers using HMDI as the coupling agent, PEG-PCL-PEG triblock copolymers were eventually obtained.

The injectable thermosensitive hydrogel was prepared by the PDMP hydrogel composite in accordance with a previously published protocol [12]. The PECE copolymer was dissolved in saline as as to attain a solution at room temperature. Then, the DDP solution and MPEG-PCL/PTX micelles were added tothe PECE solution to make a homogeneous mixture. In the course of the process, the PECE concentration was kept at 30% wt. The prepared PDMP hydrogel composite contains the following components (per ml of hydrogel): 0.3 g PECE, 0.4 mg DDP, 25 mg MPEG-PCL/PTX micelles, and the medium was normal saline. Prior to injection, the prepared PDMP hydrogel composite was sterilized using gamma sterilization (Linac) (20 Gy).

### Rheological study of the PDMP hydrogel composite

Applying an AR2000ex rheometer (TA Instruments, USA) aims to carry out Rheological study of the PDMP hydrogel composite. Rheological measurements of the PECE copolymer solution and PDMP hydrogel composite. Briefly, the samples were put between parallel plates which have a diameter of 40 mm and a gap of 1 mm. The heating rate was 2°C /min over the scope of 10 to 60°C. The storage modulus (G’) and loss modulus (G’’) were used to measure the functions of temperature. A frequency of 1 Hz and a strain of 1% were used to kept a linear viscoelastic region

### Murine tumor models and treatment

HeLa cells (1 × 10^6^/mL) were implanted in the right thigh of female BALB/c nude mice. Two weeks after injection, at the initiation of treatment, the tumor volume reached 150–200 mm^3^, which was calculated using the formula: tumor size = (length) × (width)^2^/2.

Subsequently, we assigned 48 tumor-bearing mice to one of the following four groups at random: 1, normal saline (NS); 2, PTX (5 mg/kg) and DDP (2 mg/kg) (PTX+DDP); 3, PDMP hydrogel composite (PDMP) and 4, Blank thermosensitive hydrogel (PECE); (*n* = 12 mice per group). Treatments were administered through intratumoral injection of the above-mentioned compounds on the first day that the animals were divided into groups. The intratumoral injections of drugs were given in the tumor side thigh of the mice. The drug doses for PTX (5 mg/kg) and DDP (2 mg/kg) were chosen according to previous studies, and have been shown to be effective in a rat tumor model [12, 36]. In addition, the volume of PDMP was based on the body weight of mice and was between 100 µL and 200 µL. The tumor diameter was measured every 2 days, for 100 days of treatment. After 2 days of treatment, 3 mice per group were sacrificed and their tumors were harvested for evaluation of γ-H2AX by immunohistochemistry, and ATM by western blot analysis. In addition, 3 mice per group were sacrificed after 10 days of treatment and tumor tissue was harvested for flow cytometry and immunohistochemical analysis. The remaining mice were used for observations of tumor growth and survival. All animal care and experimental procedures were conducted according to the Institutional Animal Care and Use guidelines.

### Toxicity assessment

Possible side effects of PDMP in mice were observed through monitoring of life span, weight, behavior , diarrhea , appetite, and life span. After mice were sacrificed, their organs including spleen, lung, liver, heart and kidney, and were harvested and hematoxylin & eosin (H&E) staining was performed. Sections were observed by two pathologists who were blinded to the treatment groups.

### Immunohistochemistry

Harvested tumors were fixed in 10% neutral buffered formalin solution, embedded in paraffin, and sectioned at 4μmthickness for immunohistochemistry analysis. Sections were evaluated for the expression of γ-H2AX, CD31, and CD133, and staining was carried out according to the manufacturer’s guidelines(Bioworld technology, China). Images were taken using an optical microscope (Olympus, Tokyo, Japan). For each tumor sample, the number of γ-H2AXand CD133 positive cells was calculated from 5 randomly selected fields (at 400× magnification) as the number of positive cells/total counted. Micro-vessel density (MVD) of tumor tissues was calculated as the mean value of CD31-positivemicrovessels in 5 randomly selected fields (at 400× magnification).

### Western blot analysis

Harvested tumors were homogenized and centrifuged for 10min at 4°C at 12,000 × g. The supernatant was collected and used for protein quantization with the bicinchoninic acid protein assay (BCA Protein Assay Reagent, Thermo Fisher Scientific, Rockford, IL, USA) and for western blot analysis. Forty micrograms of protein was run on a 4–20% Tris-glycine gel and transferred to 0.45 µm nitrocellulose membranes. The membranes were blocked with 5% non-fat milk in tris-buffered saline. After blocking, membranes were incubated overnight at 4°C with the primary antibody (anti-ataxia telangiectasia mutated(ATM) monoclonal antibody MAT3–4G10/8, Sigma). Next, membranes were washed three times for 10minwith Tris-buffered saline, and incubated with a peroxidase-conjugated secondary antibody for 1hat room temperature, with shaking. After incubation, the membranes were washed three times for 10 min in Tris-buffered saline at room temperature, and developed using chemiluminescence. Glyceraldehyde-3-phosphatedehydrogenase(GAPDH) was included and served as a negative control. Signals were quantified by using ImageQuant 5.0 software (Molecular Dynamics, Sunnyvale, CA, USA).

### Flow cytometry

A single-cell suspension of 1 × 10^6^ cells/mL was prepared from isolated tumor tissue and 10 mL of this suspension was added to individual testtubes. Five mL of annexin Vfluorescein isothiocyanate (FITC) and 5 mL of propidium iodide were added to each tube according to the desired analysis, such as cell cycle, apoptosis or ALDH1. Tubes were incubated on ice for 15 min in the dark, and after addition of 400 mL ice-cold 1× binding buffer the solution was gently mixed. Next, samples were analyzed using an Epics XL flow cytometer (Beckman Coulter, Miami, FL, USA).

### Statistical analysis

Statistical analysis was carried out using SPSS 17.0 software. Comparison of mean values was performed by Student’s *t*-test and one-way analysis of variance (ANOVA). Comparisons of tumor growth were performed using repeated-measures ANOVA. Tumor growth delay (TGD) was defined as the difference, in number of days, between the T4V0 of treated tumors versus untreated tumors. T4V0 represents the number of days required for the tumor to reach four times its original volume after the animals were separated into groups. Survival curves were generated based on the Kaplan–Meier survival approach. The means were considered different when *P <* 0.05, and considered significantly different when *P <* 0.01.

## SUPPLEMENTARY MATERIALS FIGURES


