# Supplementary material for: Efficient inhibition of cervical cancer by dual drugs loaded in biodegradable thermosensitive hydrogel composites
Source: Oncotarget. 2017 Dec 6;9(1):282–92. doi: 10.18632/oncotarget.22965 (PMC5787464; doi:10.18632/oncotarget.22965)
Supplement: Supplementary file 1 [file oncotarget-09-282-s001.pdf]

Efficient inhibition of cervical cancer by dual drugs loaded in biodegradable thermosensitive hydrogel composites

SUPPLEMENTARY MATERIALS

Preparation of MPEG-PCL and PECE copolymers

MPEG-PCL copolymer was synthesized by the ring-opening polymerization of ε-CL on MPEG using Sn(Oct)2 as a catalyst according to the previously reported [1]. Briefly, MPEG and ε-CL were put into a dry, glass ampule under nitrogen, and Sn(Oct)2 was added into the reaction vessel under mild agitation at 130°C for 6 hours.

The triblock copolymer (PEG-PCL-PEG, or PECE) was synthesized in two steps [2]. The MPEG-PCL diblock copolymer with a theoretical molecular weight of 1650 Da (MPEG550-PCL1100) was first prepared by ring-opening copolymerization. Then the diblock copolymer was cross-linked by HMDI producing a PECE triblock copolymer with a molecular weight approximately 3140 Da.

Preparation of MPEG-PCL/PTX micelles

The PTX-loaded micelles were prepared according to the published report [3]. Briefly, a calculated amount of MPEG-PCL copolymer with a theoretical molecular weight of 4,000 Da (MPEG2000-PCL2000)—the actual molecular weight was about 3860 Da—and PTX were completely dissolved in dehydrated alcohol. After the solvent was removed on a rotary evaporator, the mixture was re-dissolved in deionized water at 65°C and filtered through a 0.22 μm filter to obtain a clarified solution. The MPEG-PCL/PTX solution was lyophilized by freeze drying to produce the MPEG-PCL/PTX micelles powder (theoretical drug loading rate of MPEG-PCL/PTX micelles was 4%).

Preparation of PDMP hydrogel composite

The first step was to prepare the injectable thermosensitive hydrogel according to previously published protocols [4, 5]. The PECE copolymer was dissolved in normal saline to form a solution at room temperature. Next, the DDP solutions and PTX micelles were mixed into the PECE solution to form a homogeneous mixture, and the concentration of PECE was kept at 30 wt%. The prepared PDMP hydrogel composite consists of the following components: (per ml of hydrogel) 0.3 g PECE, 0.4 mg cisplatin, 25 mg MPEG-PCL/PTX micelles, and the remaining medium was 0.9% normal saline. The prepared PDMP hydrogel composite was was sterilized using linear accelerators (Linac) (20 Gy) before injection.

REFERENCES

1. Wei X, Gong C, Gou M, Fu S, Guo Q, Shi S, Luo F, Guo G, Qiu L, Qian Z. Biodegradable poly(epsilon-caprolactone)-poly(ethylene glycol) copolymers as drug delivery system. International Journal of Pharmaceutics. 2009; 381:1–18.
2. Gong CY, Shi S, Wang XH, Wang YJ, Fu SZ, Dong PW, Chen LJ, Zhao X, Wei YQ, Qian ZY. Novel Composite Drug Delivery System for Honokiol Delivery: Self-Assembled Poly(ethylene glycol)−Poly(ε-caprolactone)−Poly(ethylene glycol) Micelles in Thermosensitive Poly(ethylene glycol)−Poly(ε-caprolactone)−Poly(ethylene glycol) Hydrogel. Journal of Physical Chemistry B. 2009; 113:10183–10188.
3. Wang C, Wang YJ, Wang YJ, Fan M, Luo F, Qian ZY. Characterization, pharmacokinetics and disposition of novel nanoscale preparations of paclitaxel. International Journal of Pharmaceutics. 2011; 414:251–259.
4. Gong C, Wei X, Wang X, Wang Y, Guo G, Mao Y, Luo F, Qian Z. Biodegradable self-assembled PEG-PCL-PEG micelles for hydrophobic honokiol delivery: I. Preparation and characterization. Nanotechnology. 2010; 21: 215103-215103.
5. Gong CY, Shi S, Dong PW, Zheng LX, Fu SZ, Guo G, Yang LJ, Wei YQ, Qian ZY. In vitro drug release behavior from a novel thermosensitive composite hydrogel based on Pluronic f127 and poly(ethylene glycol)-poly(ε-caprolactone)-poly(ethylene glycol) copolymer. Bmc Biotechnology. 2009; 9:1–13.

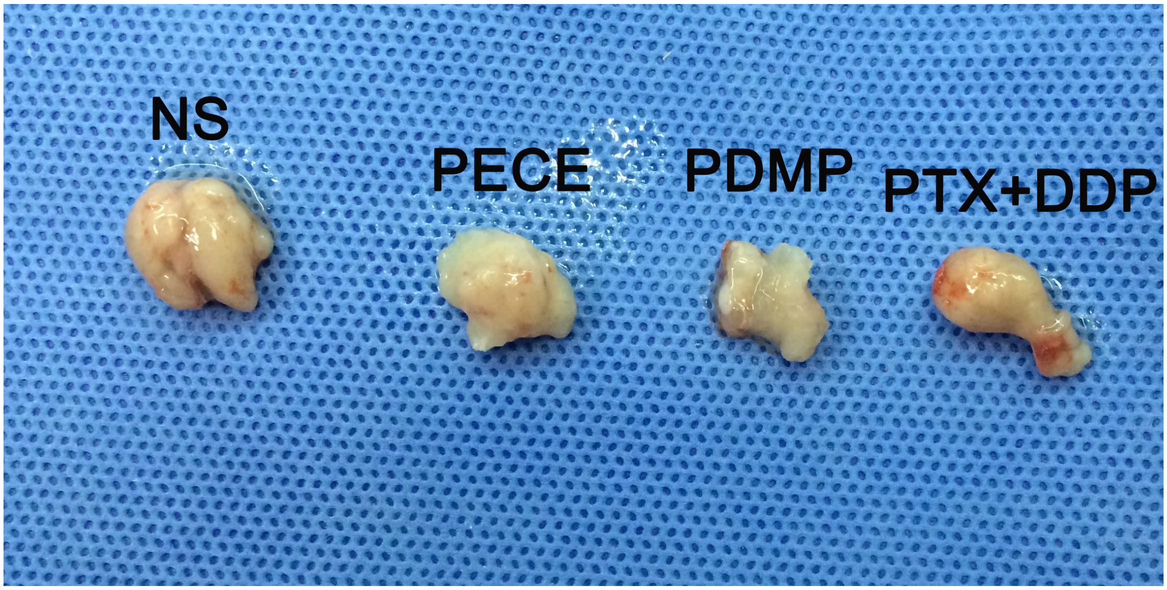

Supplementary Figure 1: The tumor size and shape of the tumor in various groups.

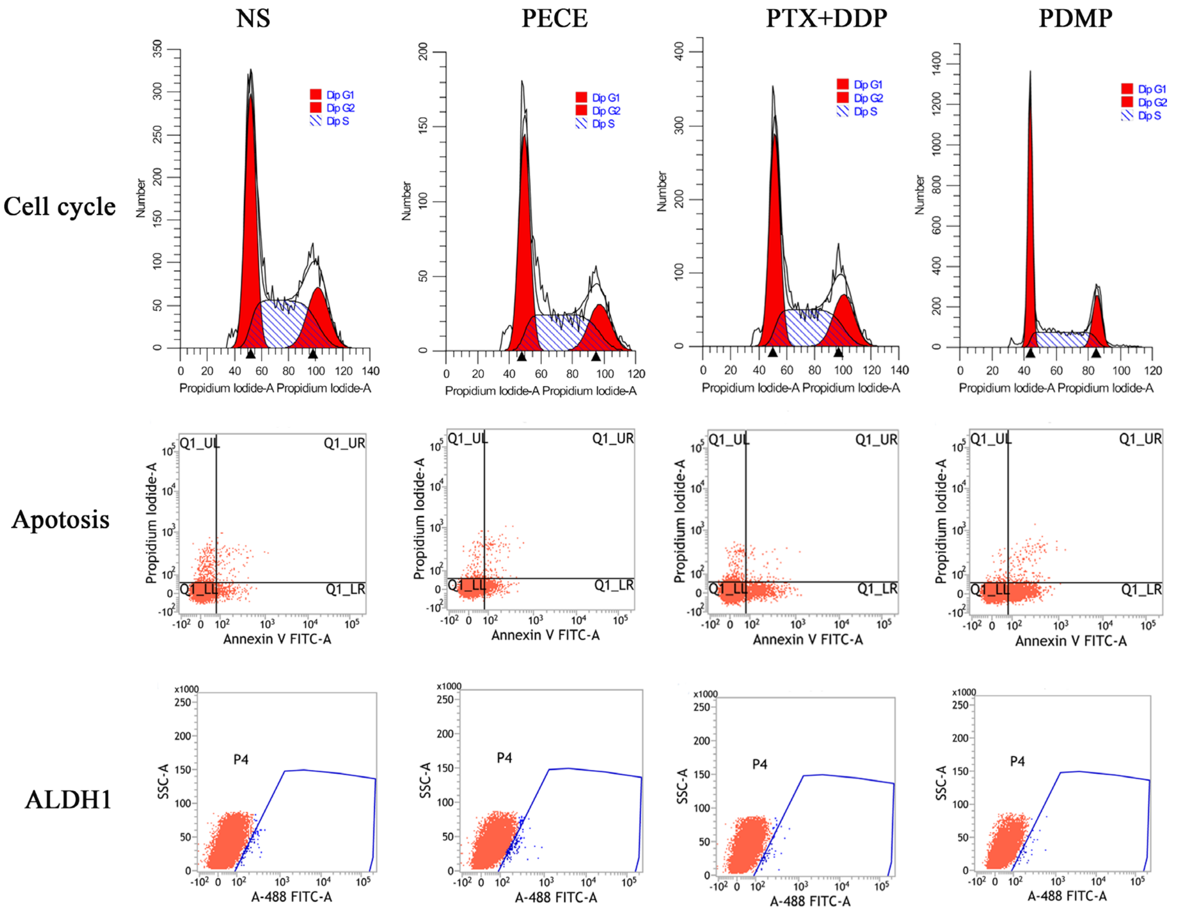

Supplementary Figure 2: Flow cytometry analysis of tumor tissue from mice that received different treatments.

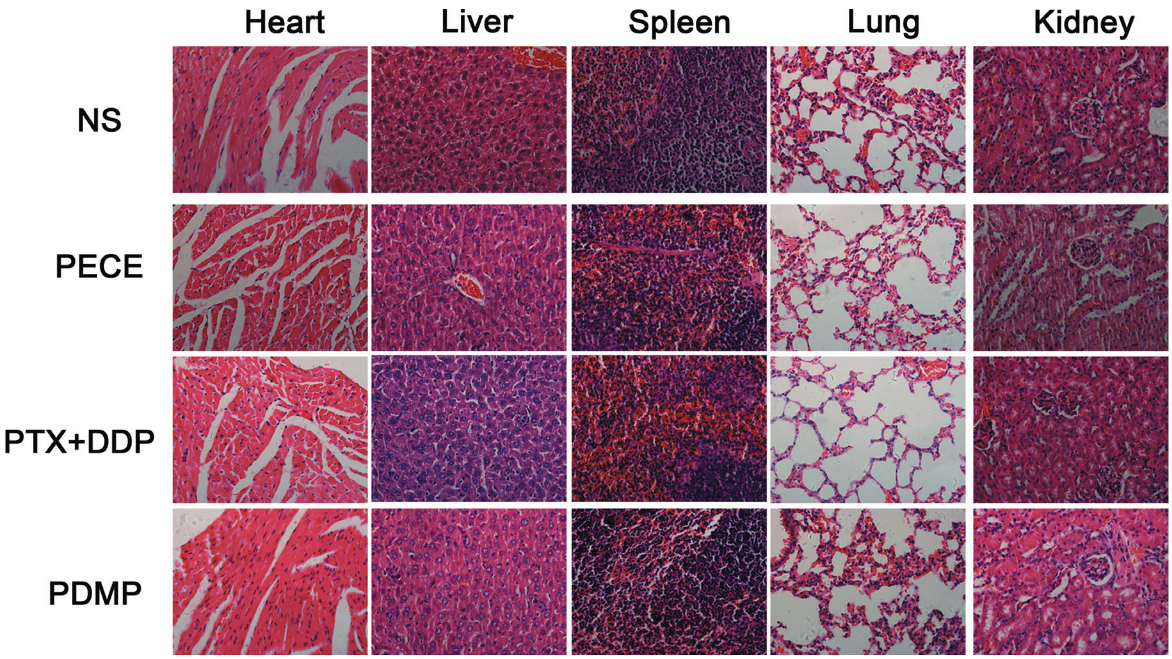

Supplementary Figure 3: Systematic toxicity in PDMP-treated mice. PTX, paclitaxel; DDP, cisplatin; PDMP, mixing MPEG-PCL/PTX micelles with DDP-loaded PECE hydrogel.
